# Loneliness, isolation and living alone associate with psychological well-being among the older adults in Taipei

**DOI:** 10.1093/geroni/igaa057.3397

**Published:** 2020-12-16

**Authors:** Hui-chuan Hsu

**Affiliations:** Taipei Medical University, Taipei, Taiwan (Republic of China)

## Abstract

Purpose: The purpose of this study was to examine the clustering of loneliness, isolation, and living alone, the risk factors and the associations with psychological wellbeing. Methods: The data were from the 2019 Taipei City Senior Citizen Condition Survey collected by face-to-face interviews, which included aged 60 and above community-based and institution-based samples. The completed sample was 3,853 persons. Loneliness, isolation, and living arrangement were analysed by cluster analysis to define the Loneliness-Isolation-Living Alone clusters. Multinomial logistic regression was used to examine the related factors to LIL clusters. Results: Four clusters of the older adults were identified and named as following: Connected (44.1%), Alone /Institutionalized (9.2%); Lonely (10.7%); and Isolated (22.0%). Compared with the Connected cluster, the Alone/Institutionalized cluster was more likely to have higher education, more IADL difficulties, more diseases , lower economic satisfaction, more likely to be males, having no spouse, and no children; the Lonely cluster was more likely to poor self-rated health, lower financial satisfaction, feeling less age-friendliness, more likely to be older, female, and no spouse; the Isolated cluster was more likely to have lower education, reported poorer self-rated health, lower economic satisfaction, and being older. The Alone/Institutionalized cluster and the Lonely cluster had higher depressive symptoms; the Alone/Institutionalized, Lonely, and Isolated clusters reported lower life satisfaction and had higher risks of cognitive impairment. Discussion: Loneliness, isolation, and living alone jointly associate with psychological health and well-being. High risk older populations may need social care and encourage social participation to promote health and wellbeing.

